# BDNF pro-peptide regulates dendritic spines via caspase-3

**DOI:** 10.1038/cddis.2016.166

**Published:** 2016-06-16

**Authors:** J Guo, Y Ji, Y Ding, W Jiang, Y Sun, B Lu, G Nagappan

**Affiliations:** 1 Neurodegeneration Discovery Performance Unit, GlaxoSmithKline, R&D China, Shanghai 201203, China; 2 Stem Cell Translational Research Center, Tongji Hospital, Tongji University School of Medicine, Shanghai 200065, China; 3 School of Medicine, Tsinghua University, Beijing 100084, China

## Abstract

The precursor of brain-derived neurotrophic factor (BDNF) (proBDNF) is enzymatically cleaved, by either intracellular (furin/PC1) or extracellular proteases (tPA/plasmin/MMP), to generate mature BDNF (mBDNF) and its pro-peptide (BDNF pro-peptide). Little is known about the function of BDNF pro-peptide. We have developed an antibody that specifically detects cleaved BDNF pro-peptide, but not proBDNF or mBDNF. Neuronal depolarization elicited a marked increase in extracellular BDNF pro-peptide, suggesting activity-dependent regulation of its extracellular levels. Exposure of BDNF pro-peptide to mature hippocampal neurons in culture dramatically reduced dendritic spine density. This effect was mediated by caspase-3, as revealed by studies with pharmacological inhibitors and genetic knockdown. BDNF pro-peptide also increased the number of ‘elongated' mitochondria and cytosolic cytochrome c, suggesting the involvement of mitochondrial-caspase-3 pathway. These results, along with BDNF pro-peptide effects recently reported on growth cones and long-term depression (LTD), suggest that BDNF pro-peptide is a negative regulator of neuronal structure and function.

Brain-derived neurotrophic factor (BDNF) is initially synthesized as precursor of BDNF (proBDNF), and endoproteolytically processed into mature BDNF (mBDNF) and BDNF pro-peptide (Figure 1a).^1, 2, 3, 4^ The role of mBDNF in neuronal development, synapse plasticity, learning and memory, and cognition is firmly established.^5, 6^ Recent research has also demonstrated that proBDNF is not an inactive precursor, instead, elicits defined biological functions. For example, proBDNF promotes apoptosis in a cell type-dependent manner,^7, 8, 9, 10^ induces neuronal spine retraction,^11^ and facilitates long-term depression (LTD) in rodent brain hippocampal slices.^12^ proBDNF is secreted by neurons in an activity-dependent manner,^2^ and elicits its function through p75^NTR^ and sortilin.^13^ Consistent with proBDNF secretion, extracellular conversion of proBDNF to mBDNF is shown to be essential for late-phase LTP (L-LTP), and this is mediated by extracellular proteases including tPA/plasmin and/or metalloproteinases MMP3, MMP7, and MMP9.^13^

It was generally believed that BDNF pro-peptide is degraded following its cleavage from proBDNF.^14^ However, study by Dieni *et al.*^15^ revealed that both mBDNF and BDNF pro-peptide were present in roughly equimolar ratio and are 10-fold more abundant than proBDNF in the hippocampus. Recent studies have shown the presence of BDNF pro-peptide in rodent brain, and membrane depolarization induced BDNF pro-peptide secretion in dissociated neuronal cultures.^15, 16, 17^ Further, BDNF pro-peptide was shown to induce acute growth cone retraction,^16^ and facilitate LTD.^17^

In this study, using a newly generated BDNF pro-peptide-specific antibody, we have unambiguously demonstrated the existence of BDNF pro-peptide in mouse brain. In addition, we report that BDNF pro-peptide levels were increased extracellularly under neuronal depolarizing conditions. Exposure of recombinant BDNF pro-peptide elicited a marked decrease in neuronal spine density at nanomolar concentrations. Interestingly, BDNF pro-peptide reduced the proportion of stubby spines, with relative increase in long, thin protruding spines. Investigation of the molecular mechanisms underlying the BDNF pro-peptide-mediated effect on dendritic spine density revealed the involvement of caspase-3, mitochondria, and cytochrome c. These results add a new dimension to the complexity of BDNF biology, and suggest that BDNF pro-peptide is a negative regulator of the structure of synapses in adult brain.

## Results

### Intact BDNF pro-peptide exists in rodent neurons

**Figure 1:**
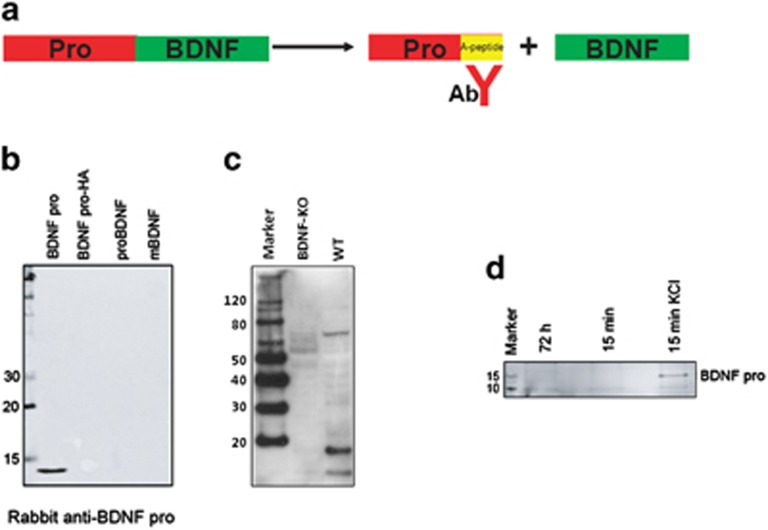
Generation and characterization of BDNF pro-peptide antibody. (**a**) Schematic illustration of proBDNF, BDNF pro-peptide and mBDNF, and the epitopes to which the BDNF pro-peptide antibody is directed. (**b**) Western blot analyses of recombinant BDNF pro-peptide, BDNF pro-peptide-HA, proBDNF, and mBDNF (10 ng each) with BDNF pro-peptide-specific antibody. (**c**) Detection of BDNF pro-peptide with BDNF pro-peptide antibody in hippocampal lysates prepared from postnatal day 7 C57/BL6 littermates – wild type and BDNF−/− mice. (**d**) Western blot analysis of endogenous BDNF pro-peptide secreted from cultured rat hippocampal neurons depolarized with or without KCl (50 mM) for 15 min. Culture media was immunoprecipitated with anti-proBDNF antibody followed by western blotting

Recent reports described the presence of BDNF pro-peptide in rodent brain using either a rabbit polyclonal (ANT-006 from Alomone Labs, Hadassah Ein Kerem, Jerusalem, Israel) or a mouse monoclonal antibody (mAb287) raised against proBDNF.^15, 16, 17^ These antibodies were raised against the sequences in the pro-domain of proBDNF, and therefore cannot distinguish proBDNF and BDNF pro-peptide *in situ*. It is also difficult to distinguish BDNF pro-peptide and proBDNF fragments with similar molecular weight. To circumvent these challenges and to be able to detect the BDNF pro-peptide, we developed an antibody specific for an epitope of the furin cleaved C-terminal end of the BDNF pro-peptide, which otherwise is unavailable in proBDNF (Figure 1a; described in Supplementary methods). This antibody specifically detected recombinant BDNF pro-peptide but not proBDNF, nor mBDNF, on western blots (Figure 1b) while the antibodies raised against respective proteins detected appropriately sized migrating proteins on the same blot when stripped and re-probed (Supplementary Figure S1a). Consistent with the design, the antibody did not detect a C-terminal HA-tagged BDNF pro-peptide (Figure 1b; detectable with anti-HA in Supplementary Figure S1a) suggesting that the free C-terminal end of the epitope is essential for antibody binding. The antibody detected BDNF pro-peptide with a sensitivity of 50 ng, and did not cross-react with NGF pro-peptide in western blot (Supplementary Figure S1b). In addition, the BDNF pro-peptide antibody detected a band corresponding to the molecular size of BDNF pro-peptide in the mouse brain hippocampal homogenates prepared from C57/BL6 wild type, but not in BDNF knockout mice (Figure 1c), indicating the specificity of this antibody.

Having confirmed the existence of BDNF pro-peptide in mouse brain, we next investigated whether endogenous neuronal BDNF pro-peptide is present in the extracellular milieu and is regulated by neuronal depolarization. BDNF pro-peptide was essentially undetectable in conditioned media collected from control cultures either 15 min or 72 h before neuronal depolarization (Figure 1d). Upon depolarization with 50 mM KCl for 15 min, BDNF pro-peptide could be reliably detected using the proBDNF antibody (Figure 1d), indicating that the extracellular levels of BDNF pro-peptide are regulated by neuronal depolarization. These results demonstrated the expression and activity-dependent extracellular regulation of BDNF pro-peptide from neurons.

### Extracellular BDNF pro-peptide alters neuronal dendritic spine density

Given that extracellular level of BDNF pro-peptide is regulated by neuronal activity, we hypothesized that BDNF pro-peptide like mBDNF may be involved in regulating synaptic structure and function. To determine the biological activities of BDNF pro-peptide, recombinant human BDNF pro-peptide was expressed and purified from *E. coli* (Supplementary Figure S2). In addition, recombinant human NGF pro-peptide (similar in molecular size to BDNF pro-peptide) was purified to be used in experiments to evaluate whether the biological activities of BDNF pro-peptide, if any, are specific to the pro-domain of BDNF or are they generic across the pro-domain of the NGF family of neurotrophins.^18, 19^

**Figure 2:**
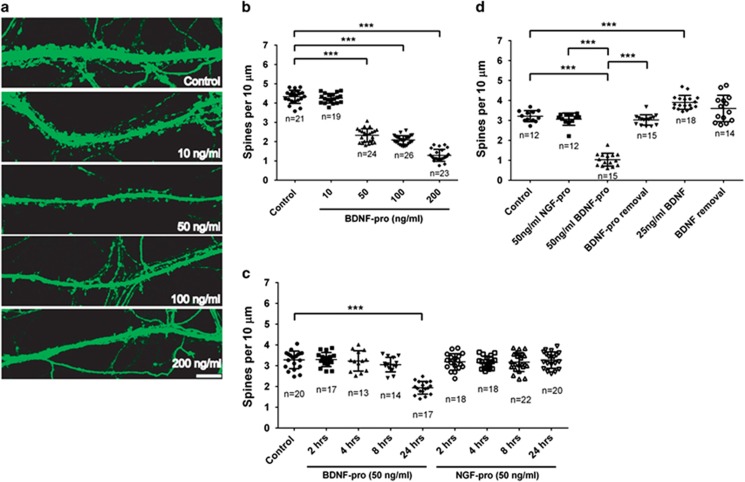
BDNF pro-peptide, but not NGF pro-peptide alters dendritic spine density in rat hippocampal neurons. (**a**) Representative images of the dendritic shafts of rat hippocampal neurons transfected with eGFP, cultured *in vitro* for ≥2 weeks and treated with different concentrations (0, 10, 50, 100, and 200 ng/ml) of recombinant human Val^66^BDNF pro-peptide for 24 h. Scale bar, 10 *μ*m. (**b**) Quantitative analysis of the dendritic spines in neurons treated with vehicle control or BDNF pro-peptide at indicated concentrations. Spines (length ≤5 *μ*m) in secondary dendritic branches were quantified and expressed as spine density (number of spines per 10 *μ*m neurite length). In this and all other experiments, a one-way ANOVA followed by Dunnett's or Tukey's test was performed to determine treatment-related differences as indicated. (**c**) Quantitative analysis of dendritic spine density in neurons treated with vehicle control or BDNF pro-peptide or NGF pro-peptide at a single concentration (50 ng/ml) for different time intervals (2, 4, 8, and 24 h); Dunnett's test (**d**) Spine density in hippocampal neurons treated with BDNF pro-peptide (50 ng/ml) was reversible, if BDNF pro-peptide is removed at least by 24 h. BDNF (25 ng/ml) increased the number of dendritic spines as expected, while its removal after 24 h did not alter dendritic spine density. '*n*' – number of secondary dendritic branches; Tukey's test. ***P<0.001

To investigate the effect of BDNF pro-peptide on dendritic spine density, rat hippocampal neurons were electroporated with plasmid expressing eGFP and grown in dissociated cultures for 16 days *in vitro* (DIV16). The cultures were treated with different concentrations (10, 50, 100, and 200 ng/ml) of recombinant human BDNF pro-peptide for 24 h. Spines (<5 *μ*m in length from the dendritic shaft) in the secondary dendritic branches were quantified manually as described in Materials and methods. Compared with vehicle-treated neurons (4.33±0.07 per 10 *μ*m, *n*=21 branches), BDNF pro-peptide treated neurons exhibited a significant reduction in spine density in a concentration-dependent manner (10 ng/ml, 4.25±0.06, *n*=19; 50 ng/ml, 2.33±0.07, *n*=24; 100 ng/ml, 2.09±0.04, *n*=26; 200 ng/ml, 1.29±0.06, *n*=23; *P*<0.001 by one-way ANOVA) (Figures 2a and b). Notably, the effect of BDNF pro-peptide on spine density was evident at 24 h but not at earlier time points (Figure 2c).

To determine the specificity of this observed effect, similar experiment was conducted using NGF pro-peptide at 50 ng/ml. This was the concentration at which BDNF pro-peptide elicited an ~50% decrease in spine density. Interestingly, treatment with NGF pro-peptide did not result in a significant change in spine density (Figure 2c; control, 3.28±0.09, *n*=20; BDNF pro-peptide 24 h, 1.94±0.07, *n*=17; NGF pro-peptide 24 h, 3.27±0.09, *n*=20; *P*<0.001, ANOVA, compared with control group).

**Figure 3:**
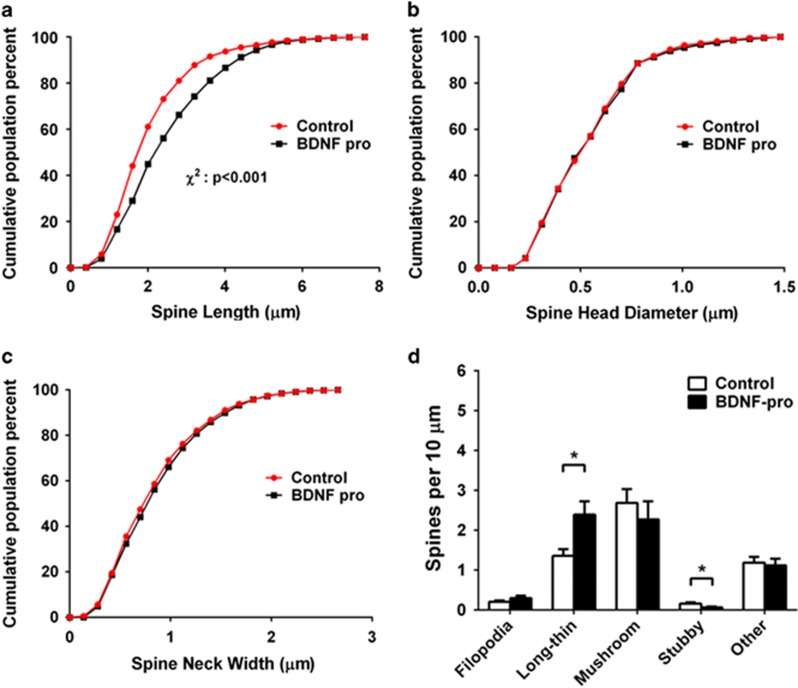
BDNF pro-peptide alters dendritic spine morphology. Neurons treated with vehicle or BDNF pro-peptide were quantified using an automated system that can clearly distinguish different types of projections from the dendritic compartment into filopodia, long-thin, mushroom, stubby, and others. A non-parametric statistical comparisons performed on the morphological analysis of the dendritic spines in neurons treated with BDNF pro-peptide (100 ng/ml) for 48 h showed an increase in spine length (*P*<0.001) (**a**) but no significant changes in spine head diameter (**b**) or spine neck width (**c**), compared with the vehicle. (**d**) Categorization and analysis of the different types of spines showed a significant reduction in stubby spines and an increase in long-thin spines. Vehicle control: *n*=96, BDNF pro-peptide: *n*=81. ‘*n*' – number of secondary dendritic branches; *P<0.05, two-tailed Student's *t*-test. Note, except this experiment, where spines are quantified using automated system, in all other experiments spines (≤5 *μ*m in length away from the dendritic trunk) were quantified manually which will preclude measuring long-thin spiny projections

To further understand the effect of BDNF pro-peptide on different types of spines, a detailed analysis of spine length (Figure 3a), spine head diameter (Figure 3b), and neck width (Figure 3c)^20^ was performed. Cumulative frequency distributions for each measure showed that BDNF pro-peptide treated neurons had a significant increase in spine length (right shift in Figure 3a), with no changes in spine head diameter and spine neck width. The cumulative frequency reflects the % population of dendritic spine projections that have a certain spine length or spine head diameter or spine neck width. For instance, 50% of the spines have an average length of 2 *μ*m in vehicle control while it is slightly longer in BDNF pro-peptide treated neurons. On the other hand, there are no treatment-related differences in spine head diameter and spine neck width compared with vehicle treatment. Assessment of the effect of BDNF pro-peptide on different types of spines revealed that BDNF pro-peptide treated neurons had a substantial increase in the number of long-thin spines with a significant decrease in stubby spines per *μ*m dendrite, with no observable changes in filopodia, mushroom, and other types of spine protrusions (Figure 3d).

Next, we tested whether the effect of BDNF pro-peptide on dendritic spine density is reversible. Hippocampal neurons were treated with 50 ng/ml of BDNF pro-peptide for 24 h, followed by a 24-h washout period before spine analysis. Identification of dendritic spines from this experiment onwards used phalloidin staining (F-actin) instead of eGFP overexpression to avoid any potential artifacts induced by either eGFP overexpression or electroporation. A cross-validation, double-blind experiment was performed to evaluate the impact of methodological differences (eGFP expression *versus* phalloidin staining). We found that the two methods yielded similar results in BDNF pro-peptide-mediated effects on spine density (data not shown). Although BDNF pro-peptide reduced dendritic spine density by ~60–70% compared with vehicle control (control 3.21±0.28, *n*=12; BDNF pro-peptide 1.03±0.32, *n*=15), NGF pro-peptide at identical concentration did not show any effect (3.06±0.31, *n*=12) as noted previously (Figure 2d). Interestingly, neurons treated with BDNF pro-peptide followed by incubation in BDNF pro-peptide free medium had similar dendritic spine density (3.03±0.26, *n*=15) as that of vehicle control (Figure 2d), indicating that the effect of BDNF pro-peptide on spine density is reversible at least by 24 h. As a positive control, addition of mBDNF resulted in an increase in spine density (3.90±0.35, *n*=18) as previously reported^21^ with negligible effect following its removal (3.60±0.66, *n*=14).

Together, these results suggest that BDNF pro-peptide when present extracellularly alters dendritic spine density, which is reversible within 24 h of induction after the removal of BDNF pro-peptide.

### Mechanistic insights into BDNF pro-peptide-mediated effect on dendritic spine density

**Figure 4:**
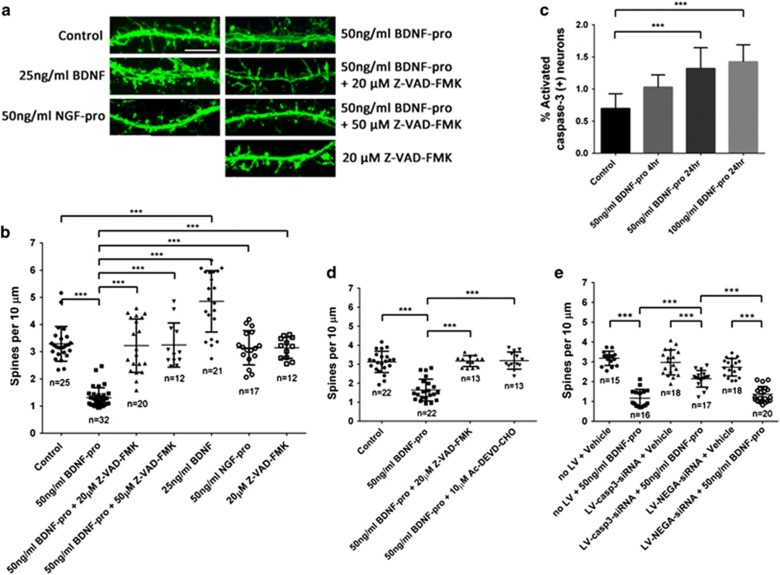
Effect of BDNF pro-peptide on dendritic spine density is mediated through caspase-3. (**a**) Representative confocal images of Alexa Fluor 488 phalloidin-stained dendritic shafts of rat hippocampal neurons treated with 50 ng/ml of BDNF pro-peptide or 50 ng/ml of NGF pro-peptide or 25 ng/ml of mBDNF, in the presence or absence of pan-caspase inhibitor Z-VAD-FMK. Scale bar, 10 *μ*m. (**b**) Quantitative dendritic spine analysis of neurons treated with BDNF pro-peptide in the presence of Z-VAD-FMK. A one-way ANOVA followed by Tukey's test showed a significant difference between neurons treated with BDNF pro-peptide in the presence or absence of Z-VAD-FMK. Z-VAD-FMK alone did not show any significant difference compared with vehicle control. (**c**) Percentage of neurons with activated caspase-3 visualized using NucView 488 caspase-3 assay as described in Materials and methods. BDNF pro-peptide increased the percentage of neurons with activated levels of caspase-3. (**d**) Pre-incubation of hippocampal neurons with caspase-3 inhibitor (10 *μ*M Ac-DEVD-CHO) completely blocked the effect of BDNF pro-peptide (50 ng/ml) on dendritic spine density. (**e**) Quantitative analysis of dendritic spines showed that BDNF pro-peptide-mediated changes in dendritic spine density were substantially reduced when caspase-3 was knocked down in neurons. Knocking down of caspase-3 in neurons by lentivirus-caspase-3-siRNA partly but significantly rescues the spine loss induced by BDNF pro-peptide, compared with that of no lentivirus transduced (no LV) and lentivirus-NEGA-siRNA transduced neurons. ***P<0.001, one-way ANOVA followed by Tukey's test

To elucidate the signaling mechanisms underlying BDNF pro-peptide regulation of spine density, we initially explored different agnostic approaches (yeast two-hybrid, cross-linking followed by pull down and mass spectrometry, protein–protein interaction on a chip) using BDNF pro-peptide as a bait. Although such an exploration identified several interacting partners, it failed to identify previously reported interacting partners, such as p75^NTR^, Sortilin,^22^ and SorCS2^16^ (data not shown). It is possible that BDNF pro-peptide induces spine loss through mechanisms independent of p75^NTR^, Sortilin, or SorCS2. We then focused on knowledge-based hypothesis-driven approach. Among several different mechanisms that are known to regulate dendritic spines, caspase pathway drew our attention given the timescale in which BDNF pro-peptide effects were observed.^23, 24^ One hour pre-incubation with a pan-caspase inhibitor Z-VAD-FMK (20 and 50 *μ*M) completely blocked the BDNF pro-peptide (50 ng/ml) effect on spine density (Figures 4a and b; control, 3.29±0.64, *n*=25; BDNF pro-peptide, 1.31±0.36, *n*=32; 20 *μ*M Z-VAD-FMK+BDNF pro-peptide, 3.22±0.98, *n*=20; 50 *μ*M Z-VAD-FMK+BDNF pro-peptide, 3.25±0.81, *n*=12), suggesting that caspase pathway is downstream of BDNF pro-peptide signaling.

**Figure 5:**
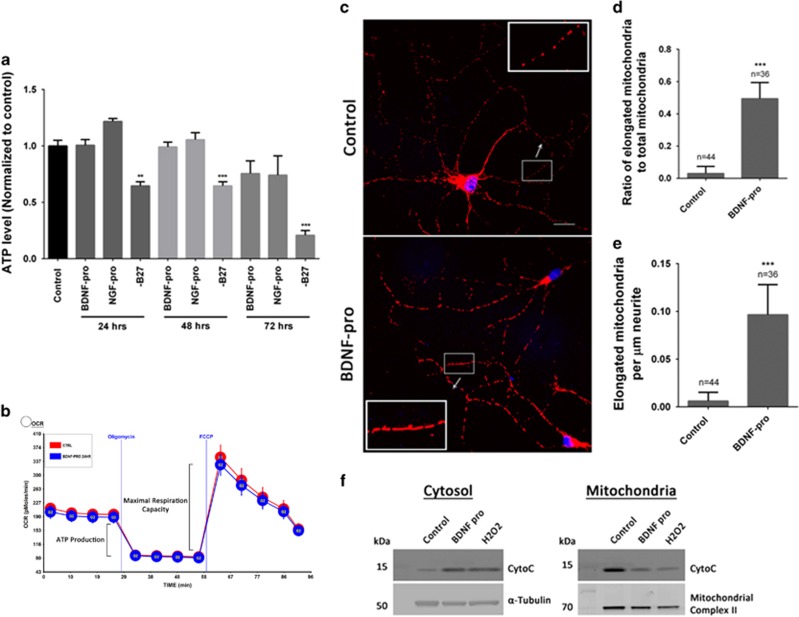
BDNF pro-peptide-mediated effects on spine density involve mitochondrial elongation and cytochrome c. (**a**) Intraneuronal ATP levels were measured using cell-titer Glo following treatment with vehicle control, 50 ng/ml BDNF pro-peptide, or 50 ng/ml NGF pro-peptide for 24, 48, and 72 h. Neurons grown in the presence and absence of B27 supplement were used as controls. Data presented are an average of three independent experiments. **P<0.01, ***P<0.001, compared with the control. (**b**) Neuronal oxygen consumption rate (OCR) measured by Seahorse X-96, following 24 h treatment with vehicle or 50 ng/ml BDNF pro-peptide either in basal conditions or following the addition of ATP synthase inhibitor oligomycin and the mitochondrial uncoupler FCCP. No significant difference in neuronal OCR, ATP production, and maximal respiration capacity following BDNF pro-peptide treatment compared with vehicle control. (**c**) Representative images of hippocampal neurons treated with vehicle control or 50 ng/ml of BDNF pro-peptide and immunostained with antibody against mitochondrial complex V enzyme (ATP synthase). Scale bar, 50 *μ*m. Inset shows higher digital magnification. (**d**) Ratio of ‘elongated' mitochondria to total mitochondria, and (**e**) the number of elongated mitochondria per *μ*m neurite length, in secondary dendritic branches in the presence and absence of 50 ng/ml BDNF pro-peptide. *n*=number of neurite branches where mitochondria were analyzed, ***P<0.001, Student's *t-*test, two-tailed. (**f**) Immunoblot analysis of cytochrome c in cytosolic and mitochondrial fractions extracted from neurons treated with vehicle control, BDNF pro-peptide (50 ng/ml) and H_2_O_2_ (0.5 mM). *α*-tubulin and mitochondrial complex II proteins were used as markers

Previous studies^23, 25, 26^ have implicated caspase-3 in regulating dendritic spine density. To investigate whether caspase-3 activity is required for BDNF pro-peptide-mediated alterations in dendritic spine density, caspase-3 enzyme activity was measured as described in Materials and Methods. Upon treatment with BDNF pro-peptide, the number of hippocampal neurons with caspase-3 activity was substantially elevated compared with vehicle (Figure 4c). Intriguingly, the increase in caspase-3 activity did not affect neuronal survival or neuronal health since the total levels of intracellular ATP in BDNF pro-peptide or NGF pro-peptide treated neurons remained unchanged up to 48 h following the treatment (Figure 5a), while withdrawal of B27 supplement from the culture medium resulted in substantial reduction in the intracellular levels of ATP within 24 h. In addition, BDNF pro-peptide did not result in any noticeable changes in dendritic morphology in these ~3-week-old neuronal cultures.

To further establish the role of caspase-3 in the BDNF pro-peptide-induced effects, caspase-3 activity was pharmacologically blocked using the caspase-3-specific inhibitor, Z-DEVD-CHO. Pre-incubation with Z-DEVD-CHO (10 *μ*M) completely abolished the BDNF pro-peptide effect (Figure 4d; control, 3.12±0.56, *n*=22; BDNF pro-peptide, 1.64±0.57, *n*=22; BDNF pro-peptide+Z-VAD-FMK, 3.17±0.29, *n*=13; BDNF pro-peptide+Z-DEVD-CHO, 3.19±0.45, *n*=13). Moreover, expression of caspase-3 was knocked down using siRNA (lentiviral transduction, LV) in rat hippocampal neurons (Supplementary Figure S3). The effect of BDNF pro-peptide was partially ameliorated in neurons transduced with siRNA targeting caspase-3 but not with scrambled siRNA (Figure 4e, S3; LV-casp3RNAi-control, 2.97±0.63, *n*=18; LV-casp3-RNAi+BDNF pro-peptide, 2.15±0.42, *n*=17; no LV control, 3.18±0.36, *n*=15; no LV+BDNF pro-peptide, 1.17±0.45, *n*=16; scrambled/negative siRNA-LV-NEGA-siRNA, 2.73±0.44, *n*=18; LV-NEGA-siRNA+BDNF pro-peptide, 1.37±0.39, *n*=20). The incomplete rescue is likely to be due to either insufficient knockdown of caspase-3 (~40% reduction; Supplementary Figure S3b) or inability to selectively identify siRNA-transduced neurons. Taken together, these results demonstrate that Caspase pathway, in particular, caspase-3 mediates BDNF pro-peptide regulation of spine density.

### BDNF pro-peptide regulates mitochondrial morphology but not its respiratory capacity

To gain insights into the molecular mechanisms, the upstream effectors (mitochondria, cytochrome c) of the classical caspase-3 pathway were investigated. First, the involvement of mitochondria in BDNF pro-peptide-mediated neuronal effects was evaluated by measuring the mitochondrial respiration rate (oxygen consumption rate, OCR) using the Seahorse method.^27^ BDNF pro-peptide did not elicit significant changes in OCR or ATP production (Figure 5b), suggesting that BDNF pro-peptide does not affect the mitochondrial oxygen consumption capacity and the steady-state levels of ATP synthesis. Lack of BDNF pro-peptide effect on ATP synthesis is also consistent with the results on assessment of neuronal health measured by intracellular levels of ATP (Figure 5a).

Second, to explore the relationship between mitochondria and the BDNF pro-peptide effects, mitochondrial morphology was investigated in the dendritic compartments using Complex V ATP synthase as a marker. Differences in the length of mitochondria were readily visible between the vehicle control and BDNF pro-peptide treated neurons (Figure 5c). Quantitative analysis revealed a marked increase in the ratio of elongated mitochondria to total mitochondria in BDNF pro-peptide treated neurons (Figure 5d; 0.494±0.017, *n*=36), compared with vehicle (0.031±0.007, *n*=44). BDNF pro-peptide treatment also dramatically increased the number of elongated mitochondria per *μ*m of neurite length (Figure 5e; BDNF pro-peptide: 0.097±0.005, *n*=36; control: 0.006±0.001, *n*=44). While this observation establishes a relationship between BDNF pro-peptide signaling and mitochondria dynamics, the significance and the physiological relevance of this finding warrants detailed investigation.

Finally, the involvement of cytochrome c in caspase-3 activation was investigated by subcellular extraction followed by western analyses to detect changes in the levels of cytochrome c in mitochondrial and cytosolic fractions. Cytochrome c levels were relatively increased in the cytosolic fractions with a concomitant decline in mitochondrial fractions derived from neurons treated with BDNF pro-peptide, compared with the corresponding fractions from vehicle-treated neurons (Figure 5f). Similar extractions from neurons treated with H_2_O_2_ (0.5 mM, 24 h) served as a positive control. These results together indicate the integrated involvement of mitochondrial dynamics, cytochrome c and caspase-3 in BDNF pro-peptide-mediated regulation of neuronal dendritic spines.

## Discussion

Like other neuropeptides, BDNF, synthesized as proBDNF, is proteolytically converted into mBDNF and BDNF pro-peptide. The functions of mBDNF and proBDNF on different neuronal processes were well studied, while the existence of BDNF pro-peptide in the brain and its function is just beginning to emerge. The pro-domain as part of proBDNF is believed to facilitate folding of BDNF^28, 29^ and involved in the interaction with Sortilin.^28, 30^ Only very recently the presence of BDNF pro-peptide and its effects on growth cone and LTD on developing neurons have been reported.^15, 16, 17^ In this study, we showed that (a) BDNF pro-peptide is present in adult neural tissues using a newly generated BDNF pro-peptide-specific antibody, and its secretion regulated by neuronal depolarization, (b) extracellular BDNF pro-peptide reduces the number of dendritic spines in mature hippocampal neurons, and (c) these effects are likely mediated through mitochondrial mechanisms, cytochrome c and caspase-3. While further studies are necessary to help elucidate the underlying molecular mechanisms, this study reveals a new role of BDNF pro-peptide in relatively mature neurons, and provides a new perspective to BDNF biology.

A previous report suggested that BDNF pro-peptide was localized to the large dense core vesicles at excitatory presynaptic terminals in the adult mouse hippocampus.^15^ However, the proBDNF antibody cannot reliably distinguish proBDNF and BDNF pro-peptide proteins. Using a newly generated anti-BDNF pro-peptide antibody which specifically detects the processed form of BDNF pro-peptide but not proBDNF or mBDNF, we now showed that endogenous BDNF pro-peptide is detectable and is intact in adult brain hippocampus. In addition, we showed that the BDNF pro-peptide levels in the extracellular milieu are neuronal activity dependent similar to that reported in previous studies.^16, 17^ Such an activity-dependent regulation of BDNF pro-peptide is consistent with the behavior of its precursor, proBDNF, and mBDNF.

Investigation of the effect of BDNF pro-peptide on the dendritic spine density in mature hippocampal neurons revealed that BDNF pro-peptide dose-dependently altered spine density with an ED50 of ~4 nM after 24 h of treatment. This effect was specific to BDNF pro-peptide, as NGF pro-peptide at similar concentrations did not have any effect on neuronal spine density. Interestingly, a detailed automated morphological analysis attributed the reduction in spine density to a reduction in number of stubby spines and an elevation in number of long-thin spines (>5 *μ*m, which are excluded from manual spine analysis). Importantly, the effect of BDNF pro-peptide on spine density is independent of neuronal viability as the levels of ATP remain unchanged. These observations are similar to that reported for the effect of proBDNF on hippocampal dendritic spines.^31^ A previous study by Anastasia *et al.*^16^ reported that BDNF pro-peptide induces neuronal growth cone collapse. It is noteworthy that this effect was observed in young neurons (DIV 3–4) while our study was carried out in relatively mature neurons (≥2 weeks). Thus, it is tempting to speculate that BDNF pro-peptide influences the formation of neuronal circuits by regulating neuronal growth cone navigation, but participates in neuronal network plasticity in the adult brain by regulating dendritic spine density in adults. In addition, the effect of BDNF pro-peptide on growth cone was reported to be Met^66^ dependent, while our study was performed with Val^66^ BDNF pro-peptide. Given that rodents do not have endogenous Met^66^ BDNF, caution must be exercised while interpreting the effect of Met^66^-BDNF pro-peptide on rat or mouse neurons.

Our study also revealed the critical role of caspase-3 pathway in BDNF pro-peptide biology. This finding is supported by (a) BDNF pro-peptide-induced increase in caspase-3 activity in neurons and (b) blockade of BDNF pro-peptide effect by pharmacological inhibition using both pan-caspase- and caspase-3-specific inhibitors, as well as silencing of caspase-3 gene expression. Previous studies have shown that the repertoire (cytochrome c, caspase-3) necessary for regulating spines are localized and functional in dendrites of 15–18 DIV rat hippocampal neurons.^24^ We now show that BDNF pro-peptide reduced spine density through caspase-3 in neurons of similar age suggesting mechanisms that negatively regulate spine density might converge on the caspase-3 pathway to exert their effect.

Our study on the role of caspase-3 in BDNF pro-peptide signaling provided some interesting insights. First, the intracellular levels of neuronal ATP and the mitochondrial respiratory capacity remained unaltered after BDNF pro-peptide treatment, despite changes in dendritic spine density. This result suggests that the effect of BDNF pro-peptide on dendritic spines is not a consequence of apoptosis; rather a specific and active mechanism in regulating spine dynamics. Second, the imaging experiments revealed an increase in number of ‘elongated' mitochondria in the BDNF pro-peptide treated neurons but not in untreated neurons. Interestingly, neuronal activity have been reported to alter mitochondrial motility and mitochondrial fission/fusion,^32^ suggesting that BDNF pro-peptide-mediated mitochondrial elongation may be directly or indirectly related to neuronal activity, which is consistent with the effect of BDNF pro-peptide on LTD.^17^ Studies have shown that mitochondrial elongation is a cell signaling response and it retains the ability to maintain ATP production, which is consistent with our observations.^33^ Third, subcellular fractionation of BDNF pro-peptide treated neurons showed a relative increase in the levels of cytochrome c in cytosol with a concomitant decrease in mitochondrial fraction. Literature evidence shows that even when cytochrome c levels are increased in cytosol, levels of cytosolic XIAP, Apaf-1, and others (apoptosome complex) need to be present at sufficient levels to initiate apoptosis.^34, 35^ Collectively, these findings implicate the involvement of the cytochrome c-caspase-3 mechanism, in addition to mechanisms responsible for regulating mitochondrial dynamics, in BDNF pro-peptide-mediated effects on dendritic spines.

What is the physiological significance of the novel function of BDNF pro-peptide (alteration of spine density) shown in this study? An obvious distinction is that the previously reported effects of BDNF pro-peptide (retraction of growth cones, regulation of LTD) were observed during early postnatal development period in which the levels of BDNF pro-peptide were relatively low (in P7 the level of BDNF pro-peptide was ~5–25% of that in 1-month-old mice^16^). It is evident that BDNF pro-peptide, like mBDNF and proBDNF, has a significant role in the establishment and regulation of neuronal networks. A recent study showed that the levels of BDNF pro-peptide are elevated (~16-fold) in the hippocampus of the post-mortem Alzheimer's brain compared with the controls with an increase in the ratio of BDNF pro-peptide to mBDNF (30 : 1). Furthermore, *in vitro* experiments using BDNF pro-peptide reported an increase in the sensitivity of SH-SY5Y neuroblastoma cells to A*β*_1-42_ cytotoxicity implicating the likely involvement of BDNF pro-peptide in neurodegenerative disease such as Alzheimer's.^36^

In summary, we have uncovered a novel biological effect of BDNF pro-peptide, a product of proBDNF cleavage, on neuronal spine density in mature hippocampal neurons. While it is well established that cleaved neuropeptides could elicit very different functions from their precursor (e.g., POMC^37^), such phenomenon has not been observed in any growth factors except BDNF. Unlike neuropeptides which are diffused or degraded relatively fast, BDNF pro-peptide and mBDNF may co-exist within similar extracellular context, and elicit respective biological effects in the same timescale. It is pertinent to determine the molecular players in BDNF pro-peptide-mediated neuronal effects. All these form the basis for future studies on the physiological and pharmacological effects of BDNF pro-peptide *in vitro* and *in vivo*, as well as the underlying mechanisms.

## Materials and Methods

### DNA construct and purification of BDNF pro-peptide

DNA sequence encoding the human BDNF pro-peptide (Val^66^) and human NGF pro-peptide up to furin cleavage site was cloned into pTXB1 vector (New England Biolab, Ipswich, MA, USA) between *Nde*I and *Spe*I sites with an extra histidine residue at the C-terminus to enhance efficient cleavage of the intein affinity tag from BDNF pro-peptide and NGF pro-peptide. Recombinant BDNF pro-peptide and NGF pro-peptide were expressed in BL21 *E. coli* and purified to homogeneity using the IMPACT kit according to the manufacturer's protocol (New England Biolab, catalog no. E6901S). The endotoxin levels of the purified recombinant proteins for neuronal treatment are <0.5 EU/*μ*g.

### Neuronal dendritic spine analysis

Following treatment of the rat hippocampal neurons (cultured *in vitro* for ≥2 weeks; either eGFP labeled or stained with phalloidin), neurons were randomly selected for taking images with Z-stacks. The maximal intensity projection was performed to generate the images for analysis. Images were coded and blinded before quantification of the spines (length ≤5 *μ*m^38^) in the secondary dendritic branches using the confocal software (Nikon-A1R NIS software, Nikon Instruments Inc., Melville, NY, USA). Following analysis, statistical evaluation, and interpretation, the group codes were unblinded to derive conclusions.

### Caspase-3 activity assay

Caspase-3 activity was assessed with the NucView 488 caspase-3 Assay Kit (Biotium, Hayward, CA, USA, catalog no. 30029) in 14 DIV rat hippocampal neurons treated with 50 ng/ml of BDNF pro-peptide for 24 h. In brief, caspase-3 activity in BDNF pro-peptide treated neurons in a 96-well plate was visualized by incubation with Hoechst 33258 and NucView 488 DNA dye conjugated to caspase-3 substrate for 30 min at room temperature. Plates were scanned in Acumen to measure caspase-3 activity by excitation at 488 nm (emission at 530 nm) and to acquire cell number (nuclei) by excitation at 352 nm (emission at 460 nm) for quantification.

### Caspase-3 knockdown in rat hippocampal culture

Lentiviral vectors expressing siRNA for caspase-3 (LV-casp3-siRNA) and scrambled siRNA (LV-NEGA-siRNA) were cloned downstream of CMV promoter, packaged, and purified by GENECHEM (Shanghai, China). Rat hippocampal neurons cultured *in vitro* for 16 days were transduced with LV-casp3-RNAi or LV-NEGA by the addition of 25 *μ*l of 5 × 10^8^ TU/ml (MOI 25). Briefly, cultured rat hippocampal neurons were incubated with LV-casp3-RNAi or LV-NEGA in neurobasal medium supplemented with 2% B27, 1% Glutamax-I, and 0.2% Penicillin-Streptomycin for 6 h at 37 °C. The culture medium containing lentivirus was replaced with neurobasal medium supplemented with 2% B27, 1% Glutamax-I, and 0.2% Penicillin-Streptomycin for 48 h at 37 °C. Caspase-3 mRNA levels were measured using real-time PCR with the forward primer 5′-CTACCGCACCCGGTTACTAT-3′ and reverse primer 5′-TTCCGGTTAACACGAGTGAG-3′. The target sequences for caspase-3-siRNA and NEGA-siRNA are 5′-GCAGCTAACCTCAGAGAGA-3′ and 5′-TTCTCCGAACGTGTCACGT-5′, respectively.

### Measuring cytochrome c in neurons

Cytochrome c in mitochondria and cytosol was measured by selective extraction using the Qproteome Mitochondrial Isolation Kit from Qiagen (Valencia, CA, USA) (catalog no. 37612). Rat hippocampal neurons (1 × 10^7^; ±50 ng/ml BDNF pro-peptide) were scraped from plates and collected by centrifugation at 200x*g* for 5 min at 4 °C. Mitochondrial isolation was performed according to the manufacturer's instructions, and 10 *μ*g total protein from the cytosolic and mitochondrial extracts was resolved on a NuPAGE 4–12% Bis-Tris gel under denaturing and reducing conditions, transferred onto nitrocellulose membrane and probed with monoclonal mouse anti-cytochrome c antibody (Abcam, Cambridge, MA, USA, 1 : 200), Complex II subunit 70 kDa Fp (flavoprotein subunit) antibody (Mitosciences, Eugene, OR, USA, 1 : 1000), and mouse anti-*α*-tubulin antibody (Sigma, St. Louis, MO, USA, 1 : 1000) followed by the corresponding secondary antibodies conjugated to IR Dye.

### Statistical analysis

Statistical analysis was performed using GraphPad InStat (GraphPad Prism 6, GraphPad Software, Inc., La Jolla, CA, USA). Multiple comparisons between groups were tested using one-way ANOVA followed by Tukey's test. The unpaired two-tailed Student's *t*-test was used to compare mean difference between two groups. Differences were considered statistically significant if *P*<0.05. All data are presented as mean±S.D. or mean±S.E.M. as indicated.

## Abbreviations

proBDNF: precursor of BDNF
mBDNF: mature BDNF
LTD: long-term depression
BDNF: brain-derived neurotrophic factor
L-LTP: late-phase LTP
DIV: days *in vitro*
LV: lentivirus
OCR: oxygen consumption rate
