# Supplementary figures and images for: BDNF pro-peptide regulates dendritic spines via caspase-3
Source: Cell Death Dis. 2016 Jun 16;7(6):e2264–. doi: 10.1038/cddis.2016.166 (PMC5143394; doi:10.1038/cddis.2016.166)

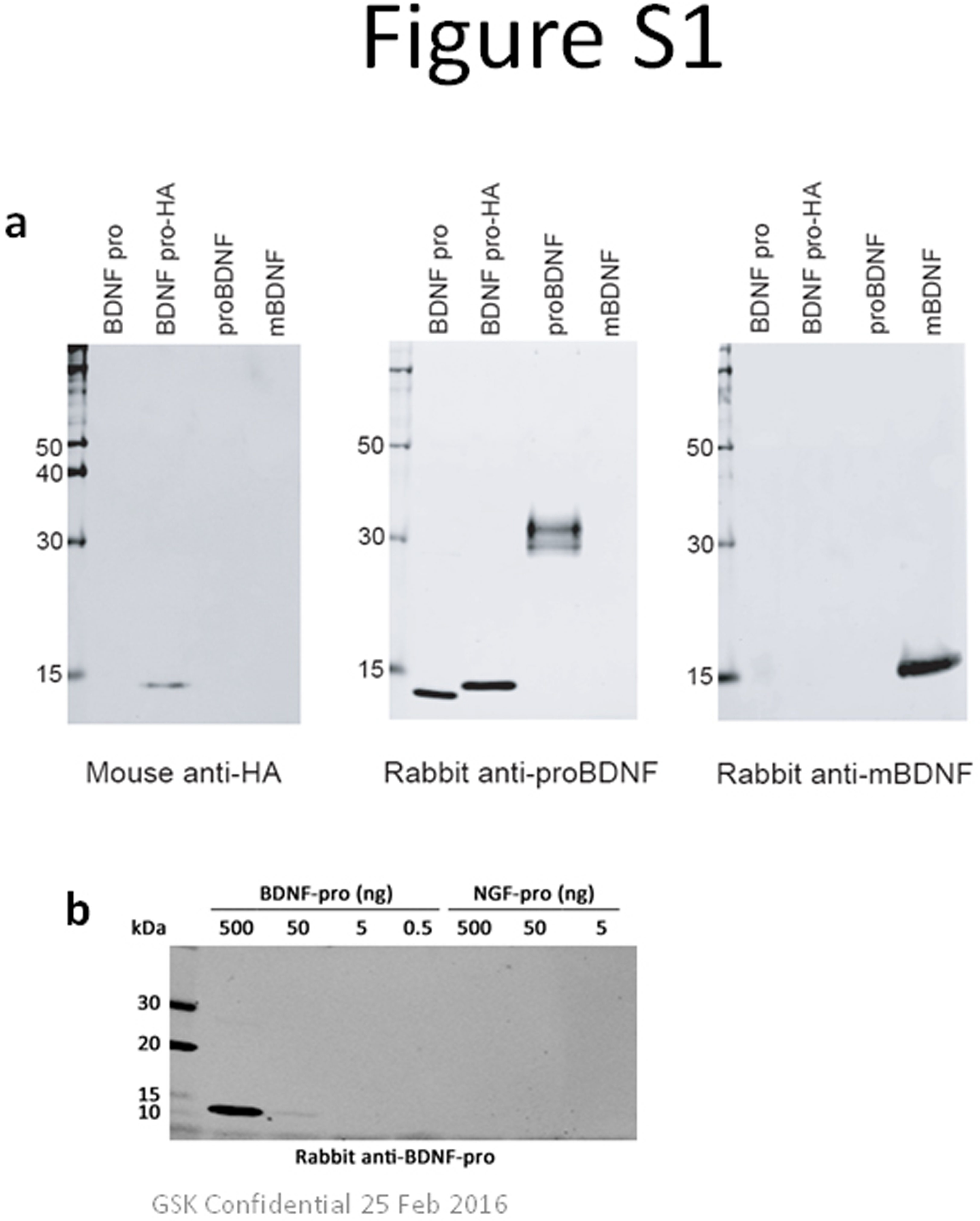

Supplement: Supplementary Figure 1 [file cddis2016166x1.tif]

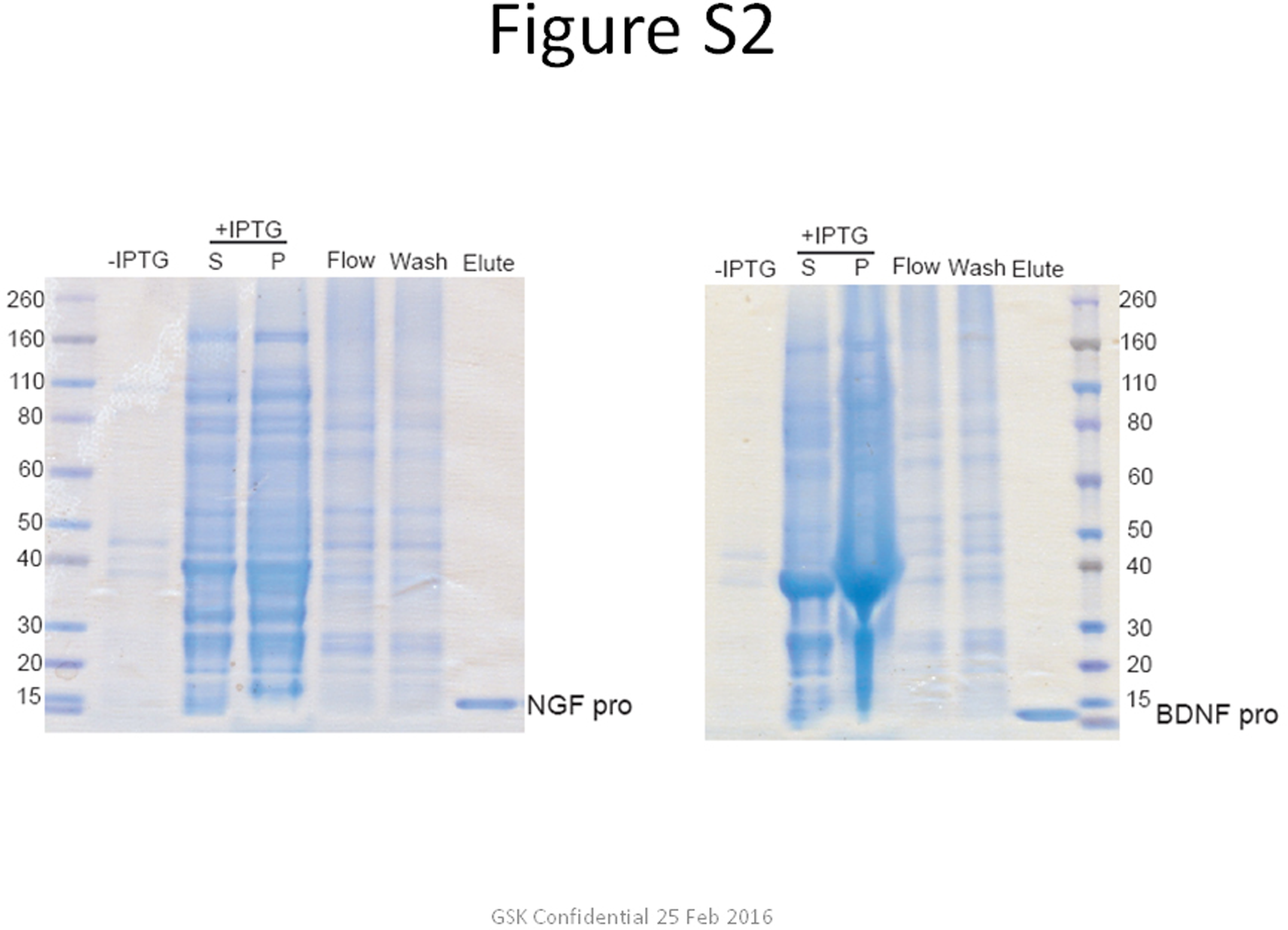

Supplement: Supplementary Figure 2 [file cddis2016166x2.tif]

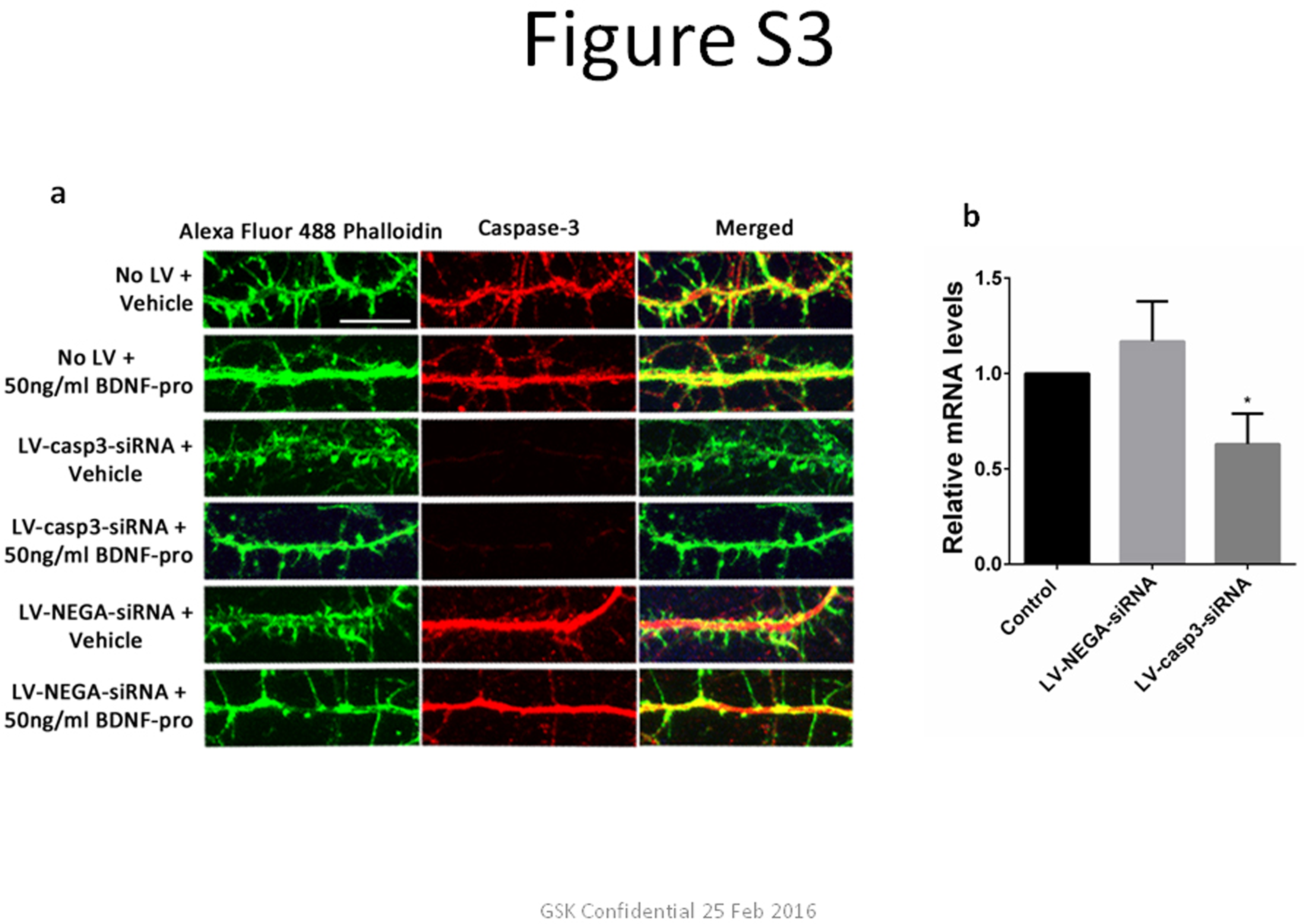

Supplement: Supplementary Figure 3 [file cddis2016166x3.tif]
