# Supplementary material for: BDNF pro-peptide regulates dendritic spines via caspase-3
Source: Cell Death Dis. 2016 Jun 16;7(6):e2264–. doi: 10.1038/cddis.2016.166 (PMC5143394; doi:10.1038/cddis.2016.166)
Supplement: Supplementary Figure Legends [file cddis2016166x4.doc]

**Supplementary Figures**

**Figure S1. Characterization of BDNF pro-peptide antibody.** (a) Western analyses of the blot shown in Figure 1b stripped and re-probed with anti-HA or proBDNF or mBDNF antibody. (b) BDNF pro-peptide antibody detects ≥50 ng BDNF pro-peptide but failed to detect even 500 ng of NGF pro-peptide.

**Figure S2. Expression and purification of BDNF pro-peptide and NGF pro-peptide.** Recombinant human BDNF pro-peptide and NGF pro-peptide were expressed and purified as described in methods. Reducing SDS-PAGE analysis of the different steps of purification visualized with Coomassie Brilliant Blue staining. –IPTG: bacterial lysate before IPTG induction; +IPTG: sample after ITPG induction; S: supernatant; P: pellet; Flow: flow through from chitin bead column; Wash: wash from the column after binding the bacterial lysate; Elute: eluate containing the purified protein.

**Figure S3. Validation of caspase-3 knock down in neurons.** (a) Caspase-3 knock down by lentiviral (LV) transduction of siRNA directed to caspase-3 and scrambled siRNA (LV-NEGA) visualized by caspase-3 antibody staining in neurons labeled with Alexa Fluor 488 phalloidin for F-actin. Hippocampal neurons with caspase-3 knock down showed significantly reduced staining for caspase-3. (b) Relative mRNA levels of caspase-3 in lentivirus-caspase-3 siRNA transduced neurons compared to untransfected control and lentivirus-NEGA siRNA transduced neurons measured by RT-PCR analysis.
